# Magnetic Resonance Microscopy at 14 Tesla and Correlative Histopathology of Human Brain Tumor Tissue

**DOI:** 10.1371/journal.pone.0027442

**Published:** 2011-11-15

**Authors:** Ana Gonzalez-Segura, Jose Manuel Morales, Jose Manuel Gonzalez-Darder, Ramon Cardona-Marsal, Concepcion Lopez-Gines, Miguel Cerda-Nicolas, Daniel Monleon

**Affiliations:** 1 Fundación de Investigación del Hospital Clínico Universitario de Valencia/Instituto de Investigación Sanitaria INCLIVA, Valencia, Spain; 2 Unidad Central de Investigación en Medicina, Universitat de Valéncia, Valencia, Spain; 3 Servicio de Neurocirugía, Hospital Clínico Universitario de Valencia, Valencia, Spain; 4 Departamento de Patología, Universitat de Valencia, Valencia, Spain; 5 Centro de Investigación Biomédica en Red de Enfermedades Respiratorias (CIBER-RES), Valencia, Spain; University of California San Francisco, United States of America

## Abstract

Magnetic Resonance Microscopy (MRM) can provide high microstructural detail in excised human lesions. Previous MRM images on some experimental models and a few human samples suggest the large potential of the technique. The aim of this study was the characterization of specific morphological features of human brain tumor samples by MRM and correlative histopathology. We performed MRM imaging and correlative histopathology in 19 meningioma and 11 glioma human brain tumor samples obtained at surgery. To our knowledge, this is the first MRM direct structural characterization of human brain tumor samples. MRM of brain tumor tissue provided images with 35 to 40 µm spatial resolution. The use of MRM to study human brain tumor samples provides new microstructural information on brain tumors for better classification and characterization. The correlation between MRM and histopathology images allowed the determination of image parameters for critical microstructures of the tumor, like collagen patterns, necrotic foci, calcifications and/or psammoma bodies, vascular distribution and hemorrhage among others. Therefore, MRM may help in interpreting the Clinical Magnetic Resonance images in terms of cell biology processes and tissue patterns. Finally, and most importantly for clinical diagnosis purposes, it provides three-dimensional information in intact samples which may help in selecting a preferential orientation for the histopathology slicing which contains most of the informative elements of the biopsy. Overall, the findings reported here provide a new and unique microstructural view of intact human brain tumor tissue. At this point, our approach and results allow the identification of specific tissue types and pathological features in unprocessed tumor samples.

## Introduction

Brain tumors affect a larger percentage of the population as lifespan increases. In general, in western developed countries, brain tumors represent between 1.4% and 2% of all neoplasms [1]. Although they represent a small proportion of all cancers, they are usually aggressive and both their mortality and their morbidity are high [1], [2]. Of all brain tumors, meningioma and glioma together account for almost 70%. Even those considered benign interfere with normal brain functions which are basic for normal activity. Survival rates among patients vary considerably depending on the type and grade of the brain tumor. In most of the cases, accurate diagnosis can significantly impact therapeutic planning and potentially improve the clinical outcome [1], [3], [4]. Diagnosis using Magnetic Resonance Imaging (MRI) is non-invasive and has high sensitivity, but only achieves between 60 and 90% accuracy depending on the tumor type and grade [5], [6]. The current gold standard characterization of a brain tumor is by histopathological analysis [7]. Histopathological analysis is the standard procedure routinely used to reveal the amount of necrosis, proliferative regions, collagen and vascularity within the tumor area. Despite worldwide efforts to standardize tumor evaluation criteria, the morphological classification of some human brain tumors is subject to interpretation. This uncertainty comes, among others, from the intra and inter-biopsy morphological heterogeneity of tumor tissue. In addition, the descriptive nature of morphology-based histopathology raises some discrepancies among experts [8], [9]. In addition, potential regions of interest inside the tissue or preferred orientation for histopathology slices are difficult to determine before processing the sample.

High resolution MRI (resolution lower than 100 µm) is called Magnetic Resonance Microscopy (MRM) [10], [11], [12]. MRM of excised tissue provides a detailed functional and anatomical picture of identical nature to those obtained by MRI and of higher resolution, which may be used as a bridge between clinical MRI and histopathology [13], [14]. There are some examples of the MRM characterization of excised tissues [15], [16] suggesting the applicability of the technique to human samples. However, up to date only Shenkar et al have applied MRM to the study of human excised tissue demonstrating the capabilities of MRM to detect microstructural patterns in human cerebral cavernous malformation [13]. High-field in vivo MRI may become possible in the near future and the potential for applying this technique in the evaluation of brain tumors would be very high. The use of MRM to study human brain tumor tissue may provide new microstructural information on brain tumors, useful for better characterization and better interpretation of future in vivo high field studies.

The aim of this study was the characterization by MRM of structural and morphological features of the brain tumor, such as calcifications, collagen patterns or presence of blood, which affect the MR signal in the different imaging sequences. We present here a complementary tool to the standard histopathology of tumor biopsies that may help in selecting preferential slicing during histology. With this purpose, we performed MRM imaging and correlative histopathology in 30 tissue samples from brain tumor patients. The correlation between MRM and histopathology images allowed the determination of MRI parameters for critical internal microstructures of the tumor.

## Methods

### Brain tumors samples

**Figure 1:**
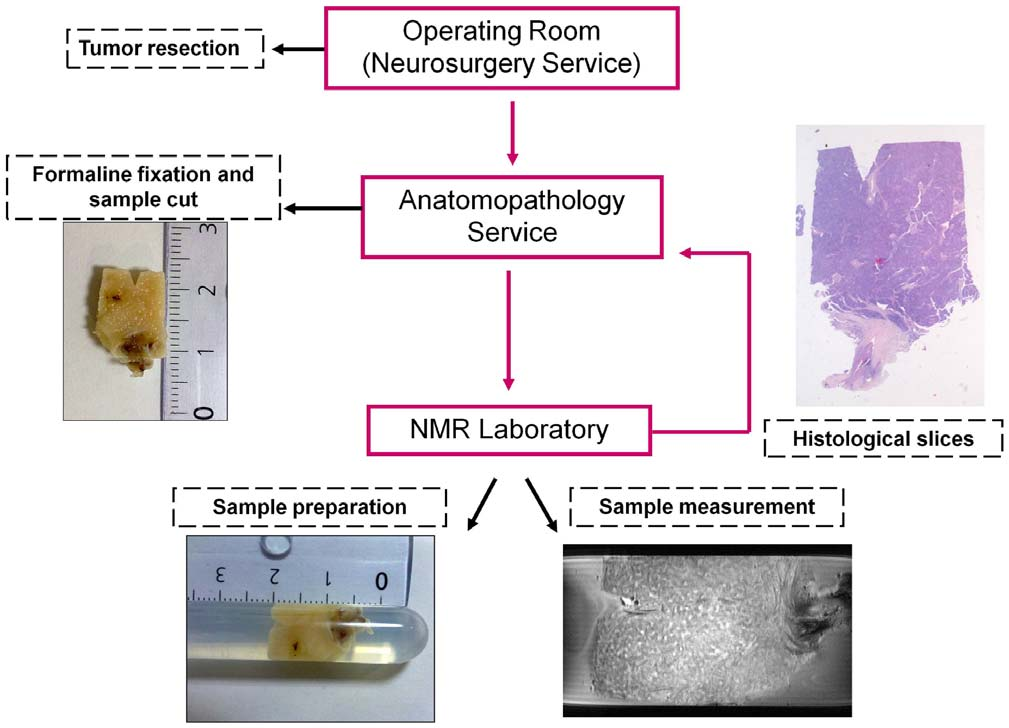
Scheme showing the protocol followed in this study. Tissue samples coming from the surgery room are fixed at the Anatomopathology Department. There, a reference cut is performed on the sample for geometrical referencing purposes. The sample is then submerged in an agarose matrix in the MRM tube at the MR Imaging lab to minimize sample motion and magnetic susceptibility artifacts. The MRM tube containing the sample is then introduced in the MR 14 Tesla magnet and MRM images are obtained at high resolution. Finally, the agarose matrix is dissolved and the samples are washed with formalin and embedded in paraffin for histopathology analysis at the Anatomopathology Department.

Brain tumor samples were obtained during surgery from patients already diagnosed with brain tumor by conventional MRI by the Neurosurgery Department and Anatomopathological Department of the Hospital Clínico Universitario de Valencia. Tissue samples used in this study included 19 samples from meningioma patients and 11 samples from glioma patients. Patients were between 25 and 92 years old and included 16 women and 14 men. This study was conducted according to the principles of the Declaration of Helsinki and was reviewed and approved by the local ethics committee. Written informed consent was obtained from all patients. All data were anonymized before analysis. Tumors were classified according to the 2007 WHO Histological Classification [7]. Three additional cavernoma samples and one additional sample from control brain tissue, obtained during the autopsy of an individual dead by no cancer related reasons, were included in the study for comparison purposes, for calibration of MRM sequences and for checking consistency of our approach with respect to previous studies [13]. All samples ranged in size from 1 to 1.8 cm and were rinsed in 4% formaldehyde for at least one month before imaging. Previous studies show that artifacts detectable by MRI are significant only after postmortem fixation periods longer than 5 years [17]. All our samples were fixed for a period between 1 month and 12 months. Before imaging a wedge was made (the reference wedge) on one side of the samples for geometrical referencing and for geometrical correlation between MRM images and histopathology images. The whole protocol for sample handling and data obtaining has been outlined in Figure 1.

### Magnetic Resonance Microscopy

Just before MRM, samples were submerged in a gel matrix (agarose 2.5%) and placed inside the imaging tube (1 cm diameter, Cortecnet, France). Magnetic resonance images were acquired at room temperature (25°C). High spatial resolution images were acquired in a 14 Teslas vertical axis imager (Bruker-AVANCE 600 system, 600 MHz proton frequency, Bruker Biospin GmbH, Rheinstetten, Germany) equipped with a 10 mm microimaging ^1^H coil tuned to the appropriate proton frequency. Maximal gradient strength was 210 gauss/cm (80% of maximum nominal strength). Two-dimensional multi-slice spin-echo images were acquired with different combinations of repetition time (TR) and echo time (TE) for obtaining different image contrasts. Slice thickness was 500 µm and the in-plane spatial resolution achieved was up to 35 microns per pixel depending on the sequence used. Matrix resolution was 512×512 pixels. Our standard protocol for imaging lasted 8 hours and consisted of T1- and T2-weighted images and proton density with the following TR/TE values: T1-weighted image, 500 milliseconds/10 milliseconds; T2-weighted image: 4000 milliseconds/60 milliseconds; proton density (PD) image: 4000 milliseconds/10 milliseconds. Gradient echo images were also acquired to obtain T2*-weighted images with TR/TE values of 1500 milliseconds/15 milliseconds. We also acquired T2-multiecho sequences (TR of 2000 milliseconds and variable TE ranging from 10 to 160 milliseconds) and diffusion weighted images (TR 1500 milliseconds, TE 15 milliseconds and b-values of 30, 49, 70, 97, 183, 298, 518, 822, 1055, 1318, 1611, 1933, 2284, 2665, 3515 and 4483 s/mm^2^) both at a matrix resolution of 256×256. The measurement system allows a nearly free choice of slice selection and orientation. We selected a slice orientation in such a way that the reference wedge was always at the topmost part of the slice and provided the largest slice image area.

### Histopathological analysis

Once MRM images were obtained, the agarose matrix was removed and the sample rinsed in 4% formalaldehyde. Then, the sample was paraffin-embedded, sliced (4 microns thickness) and examined histologically using hematoxylin and eosin (HE) dye. Histological slicing orientation was chosen to match the MRM images using the reference wedge and the maximal sample area criteria.

### Data analysis

R2 (which is 1/T2) and ADC maps were calculated in each pixel of the images from the multi-echo multi-slice image sequence and from the diffusion weighted multi-b-value image sequence, respectively. All images were converted to DICOM standard format and subsequently processed using MATLAB in-house scripts. Exponential fitting was made and thus, R2 map and ADC map were calculated for all samples in all pixels. Regions of interest (ROIs) and subsequent analysis were selected by expert neuropathologists based on the correlation between histological images and MRM images (Table 1). Relative intensity was expressed as the mean intensity in the selected ROI relative to the mean intensity of a fixed region in the agarose matrix. R2 and ADC values for selected ROIs are expressed as mean and standard deviation in Table 2.

**Table 1:** Relative MRM intensity of selected ROIs in brain tumor tissue.

	T1- image	T2- image	T2*^-^image	PD-image	R2 map	ADC
Collagen	**-**	**-**	**-**	**-**	**+**	**++**
Edema	**- -**	**++**	**++**	**+**	**++**	**++**
Vasculature	**- -**	**- -**	**- -**	**-**	**- -**	**+++**
Calcium	**- - -**	**- - -**	**- - -**	**- - -**	**++**	**- - -**
Hemorrhage	**+**	**- - -**	**- - -**	**- -**	**- - -**	**- -**
Necrosis	**-**	**- -**	**-**	**- - -**	**++**	**++**

Qualitative MRM region of interest (ROI) signal intensity trend relative to dominant tissue signal intensity for different areas as selected by expert pathologists and defined by correlation with respect histopathology images.

**Table 2:** MRM parameters for different microstructural elements in brain tumor tissue.

	I_T1_/I_T1ag_	T2 (ms)	I_PD_/I_PDag_	I_T2*_/I_T2*ag_	ADC (ms/s^−1^)
Predominant tissue (transitional meningioma)	1.78±0.15	24.2±1.0	1.31±0.11	0.85±0.05	0.69±0.03
Predominant tissue (meningiothelial meningioma)	1.83±0.14	34.7±1.4	1.52±0.09	1.14±0.04	0.94±0.12
Peritumoral region (oligodendroglioma)	1.26±0.14	31.2±0.8	1.07±0.11	0.97±0.05	0.46±0.04
Necrosis (glioblastoma multiforme)	1.58±0.25	25.2±1.4	1.27±0.20	1.08±0.08	0.97±0.08
Vasculature (meningothelial meningioma)	1.39±0.06	35.8±2.5	1.29±0.02	1.08±0.05	1.29±0.13
Collagen (transitional meningioma)	1.40±0.06	22.6±1.6	1.19±0.04	1.08±0.01	1.55±0.07
Collagen (oligodendroglioma)	1.60±0.03	21.6±0.7	1.35±0.02	1.19±0.04	1.0±0.1
Hemorrhage (gliosarcoma)	1.68±0.24	11.9±0.2	0.65±0.09	0.10±0.02	0.16±0.05
Calcifications (gliosarcoma)	1.51±0.20	16.9±0.1	0.62±0.12	0.064±0.002	0.25±0.06
White matter (control tissue)	2.05±0.14	29.0±1.4	1.04±0.07	0.49±0.05	0.10±0.01
Gray matter (control tissue)	3.27±0.26	27.9±1.4	1.80±0.15	0.97±0.04	0.24±0.01

T2 relaxation times and ADCs of different region of interest (ROI) as selected by expert pathologists and defined by correlation with respect to histopathology images. A T2 and apparent diffusion coefficient (ADC) value was calculated for each pixel in the selected ROI by exponential fitting of the intensity curves of the corresponding image sequences (T2-map and ADC-map). T2 and ADC values are expressed as mean and standard deviation for all the pixels in a selected ROI.

## Results

**Figure 2:**
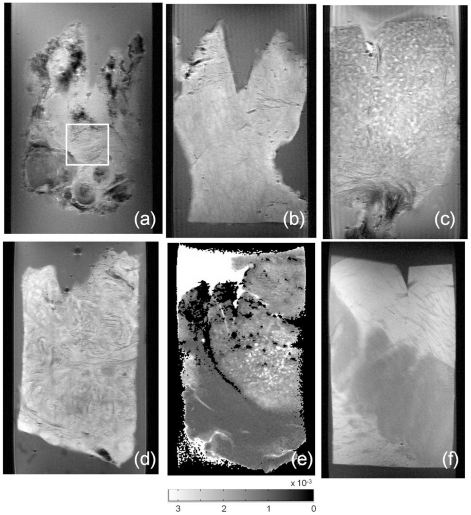
Magnetic Resonance Microscopy (MRM) images at 14 Tesla and 25°C of different human brain excised tissue showing different structural details in the different image sequences and brain tissues. A) Proton density spin echo image of a human cerebral cavernous malformation (CCM) lesion biopsy showing the potential honeycomb region enclosed in a white square; B) T2-weighted spin echo image of a human fibroblastic meningioma brain tumor; C) T2-weighted spin echo image of a human meningothelial meningioma brain tumor biopsy; D) T2-weighted spin echo image of a human gliosarcoma brain tumor biopsy; E) ADC map of a human oligoastrocytoma showing the change in water diffusion between the tumoral (high ADC) and peritumoral (low ADC, white arrows) regions; F) T1-weighted spin echo image of human healthy brain showing the differences between gray (hyper-intense) and white (hypo-intense) matter.

**Figure 3:**
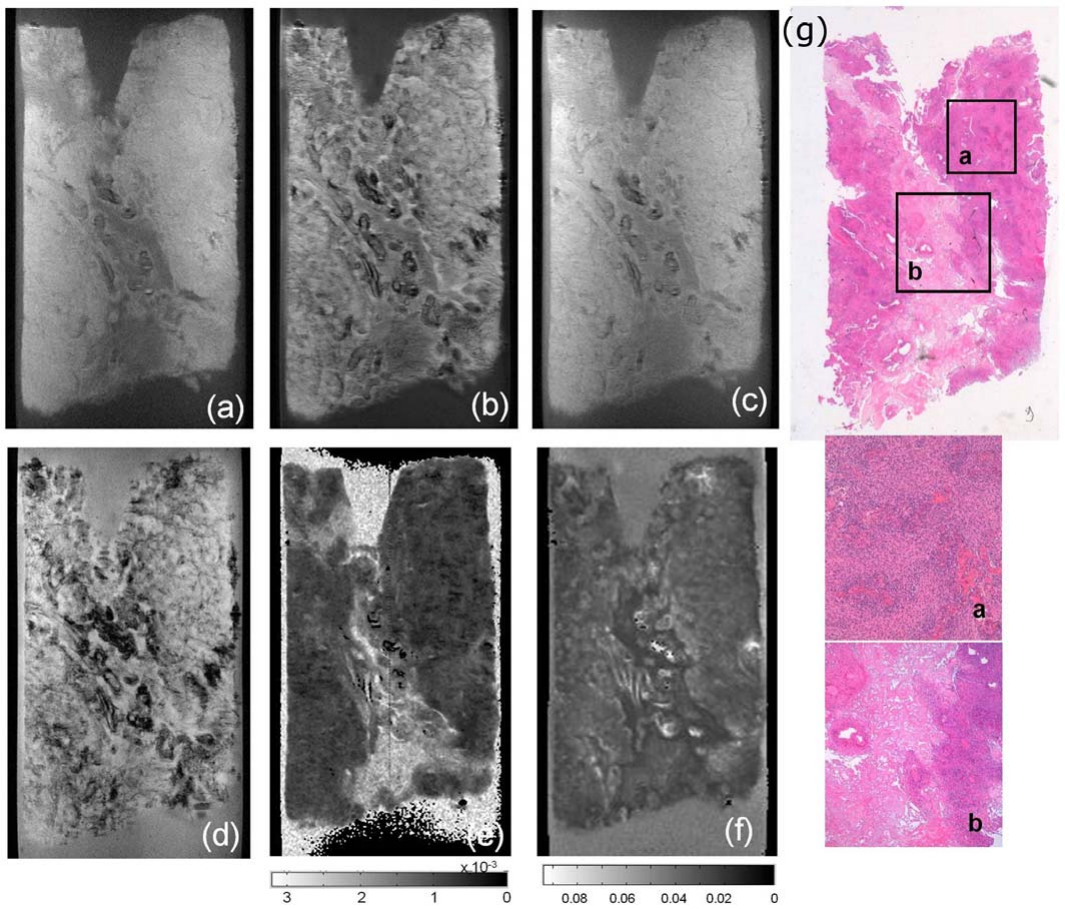
Magnetic Resonance Microscopy (MRM) images at 14 Tesla and 25°C of human meningioma brain tumor sample showing the different contrasts obtained using different image sequences (A–F) and compared to correlative histopathology (G). A) T1-weighted spin echo image; B) T2-weighted spin echo image; C) T2*-weighted gradient echo image; D) proton density weighted image; E) Apparent Diffusion Coefficient (ADC) map calculated from multi-slice multi-b-values image sequence; F) R2 map calculated from multi-slice multiecho image sequence; G) Hematoxylin and eosin (HE) stain at low magnification power of human meningioma brain tumor sample with two zoomed regions showing microhemorrhages (a) and collagen pattern (b).

MRM of brain tumor biopsies provided images with high contrast and high geometrical resolution (35 to 40 microns). Examples of MRM images for some brain tumors, brain lesions and normal brain are shown in Figure 2. All MRM images acquired and correlative histopathology are shown in the supplementary material. The brain tumor tissue images show good contrast with respect to the agarose matrix. Samples studied showed heterogeneous microstructure, including blood vessels, collagen, necrotic foci, cysts areas and calcifications. In general, tumor samples images exhibited high structural detail. MRM allowed, for example, the observation of peritumoral regions in glioma (Figure 2D), the distinction between gray and white matter in healthy brain tissue (Figure 2E), the observation of microvessels in meningioma (Figure 2C) or the identification of caverns in cavernomas (Figure 2A). In this sense, we obtained MRM images for three cavernomas in order to compare our results with those reported by Shenkar et al and therefore validate our sample preparation protocol. Similarly to previous studies, cavernoma MRM images exhibited a highly heterogeneous microstructure with ‘bland’ regions which have large caverns larger than 500 um and ‘honeycombed’ regions with smaller caverns and/or capillaries [13]. These results suggest that, as expected, the agarose matrix does not alter the sample microstructure. ADC and R2 maps provide additional information to determine different regions in cavernomas. The histopathology study ensures the correct interpretation of the different MRM regions. In our study, we used MRM for microstructural characterization of two types of human brain tumor samples: gliomas and meningiomas. The acquisition of different images on each sample and the comparison with respect to histopathology (Figure 3) allowed a better characterization of the microstructural elements present in the samples. Table 1 shows the MRM characteristics of differential regions within these human brain tumors.

**Figure 4:**
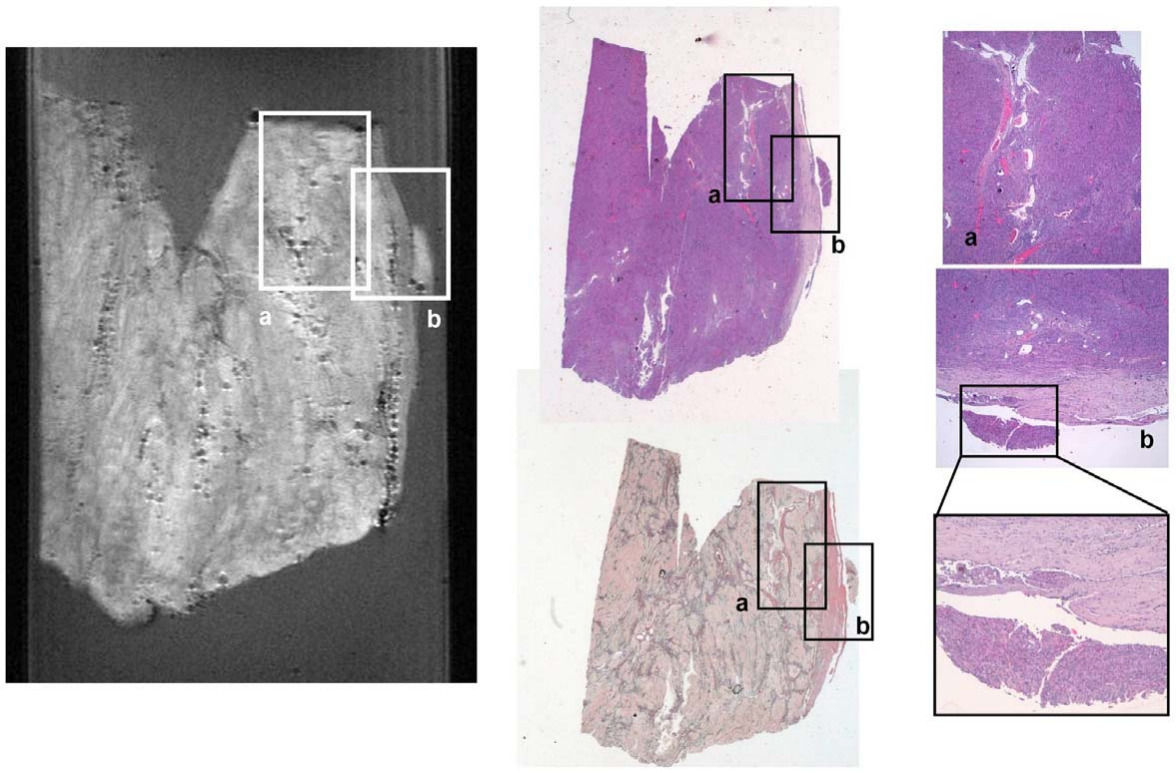
Magnetic Resonance Microscopy (MRM) T2-weighted image at 14 Tesla and 25°C (right), correlative histopathology hematoxylin and eosin (HE) dye (top middle and left) and correlative histopathology Gomori's trichrome dye (bottom middle) of human transitional meningioma tumor sample. Regions of interest containing vasculature (a) and collagen in duramadre (b) have been highlighted and zoomed in.

**Figure 5:**
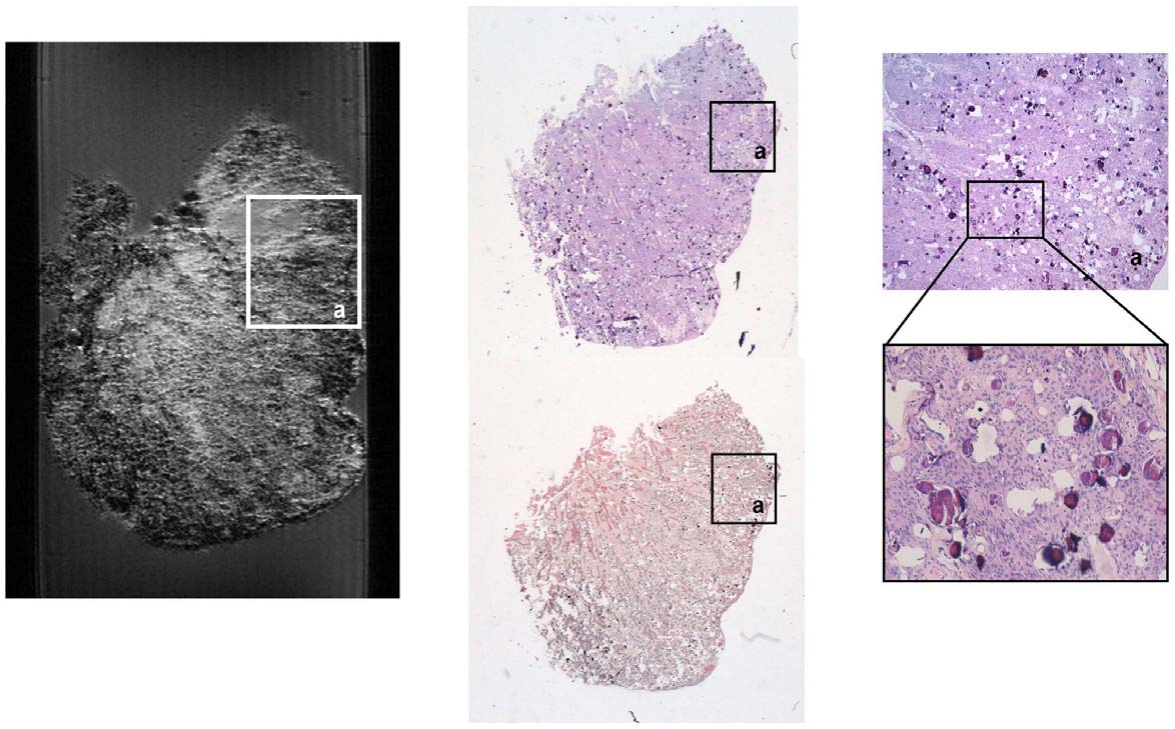
Magnetic Resonance Microscopy (MRM) T2-weighted image at 14 Tesla and 25°C (right), correlative histopathology hematoxylin and eosin (HE) dye (top middle and left) and correlative histopathology Gomori's trichrome dye (bottom middle) of human transitional meningioma tumor sample. Calcifications and psammomas bodies (a) can be seen spread all over the image as hyper intense spots in the MRM image and dark spots in the histopathology stain.

In general, MRM images of human meningioma biopsies show large areas of uniform signal intensity with some hypo-intense elements spread over the entire sample (Figure 3, 4 and 5). Meningioma tissue appears at higher intensity than the agarose matrix in all images acquired. Due to the broad typology of meningioma, microstructural elements observed in the different meningioma samples are diverse. Among the different types of meningiomas, fibroblastic and transitional meningiomas show large areas of homogeneous signal intensity (Figures 2B and 4) whereas microcystical and meningiothelial meningioma exhibit some hypointense regions, which correspond to microcysts, vessels and nodular patterns (Figures 2C and 5). Collagen structures, which show the most extensive signal intensity heterogeneities in MRM images of meningioma, have different appearance in the different meningioma subtypes. The comparison between MRM images and Gomori's trichrome stains allows the detection and correlation of collagen bands in both images. Gomori's trichrome stains green or blue connective tissue and collagen. This stain allows triple staining in one step [18]. Collagen in fibroblastic and transitional meningiomas usually appears as hypo-intense grooves in T2-weighted images and slightly hypo-intense regions in proton density and T1-weighted images. On the other hand, collagen in microcystical meningiomas appears as large slightly hypo-intense bands in T2-weighted images. Meningiothelial meningiomas also display a variety of T2 and T2*-weighted hypo-intense microregions. Microvasculature, as determined by correlative histopathology, appears highly hypo-intense in these images.

**Figure 6:**
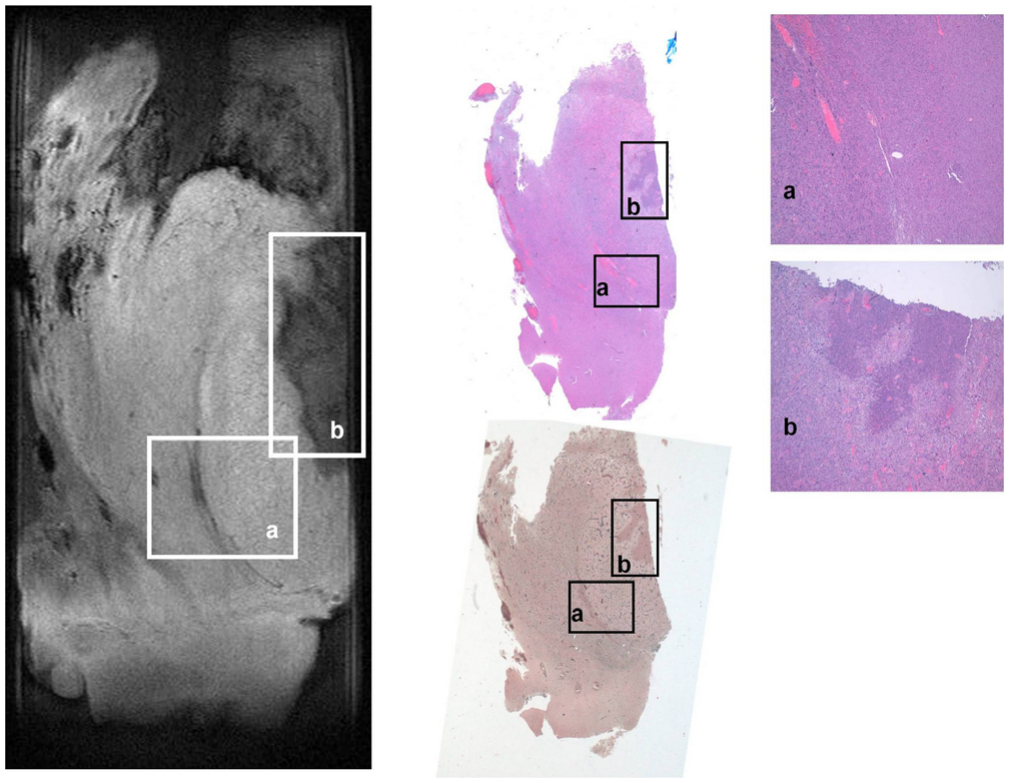
Magnetic Resonance Microscopy (MRM) T2-weighted image at 14 Tesla and 25°C (right), correlative histopathology hematoxylin and eosin (HE) dye (top middle and left) and correlative histopathology Gomori's trichrome dye (bottom middle) of human glioblastoma multiforme tumor sample. Region of interest containing the tumor infiltrative border, hemorrhage and congestive vasculature (a) and region of interest containing tumor necrotic foci (b) have been highlighted and zoomed in.

**Figure 7:**
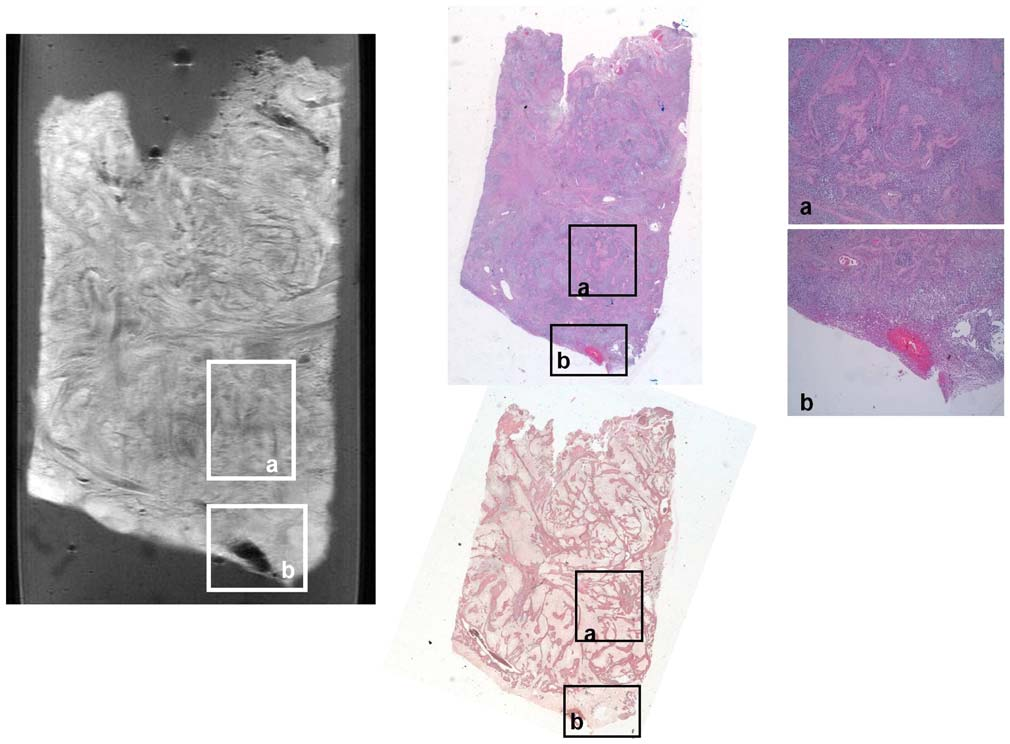
Magnetic Resonance Microscopy (MRM) T2-weighted image at 14 Tesla and 25°C (right), correlative histopathology hematoxylin and eosin (HE) dye (top middle and left) and correlative histopathology Gomori's trichrome dye (bottom middle) of human gliosarcoma tumor sample. Regions of interest containing characteristic sarcomatous vascular and collagen pattern (a) and hemorrhage (b) have been highlighted and zoomed in.

In contrast to meningioma, MRM images of human glioma tissue show signal intensity areas of larger variability appearing in MRM images as more heterogeneous (Figures 2, 6 and 7). Nevertheless, all glial tumors appear with higher intensity than agarose exhibiting some hypo-intense elements. T1 and T2-weighted MRM images of human glial tumors show different hypo and hyper-intense regions organized in very different patterns in the samples studied. The combination of T1 and T2-weigthed images together with ADC maps reveals some microstructural elements otherwise hidden. As expected, glioblastoma multiforme, which is a highly heterogeneous tumor, shows large areas of necrosis and angiodysplastic microvessels. In our glioblastoma multiforme samples we also observed, when present, tumor infiltrative border as a slightly hypointense band in T2-weighted images (Figure 6). Blood breakdown products appear as foci that are highly hypo-intense in T2 and T2*-weighted images and hyper-intense in T1-weighted images. Microvessels show hypo-intensity in T2 and T2*-weighted images and hyper-intensity in proton density and T1-weighted images. If present, calcium deposits appear hypo-intense in all imaging protocols. Necrotic foci show hypointensity in all imaging protocols, with the exception of the ADC maps, suggesting increased water diffusion. Interestingly, peritumoral region only exhibits differential signal intensity in ADC images (Figure 2E) with lower water diffusion. Many of these microstructural elements also appear in other glial tumors. Oligodendrogliomas show large regions with homogeneous intensity and some smaller regions with higher concentration of hypo-intense elements. Gliosarcoma (Figure 7), which is a less common glial tumor with very characteristic microstructural features, shows high contrast with respect to agarose with highly structured patterns and microelements spread all over the image. Collagen forms large bands slightly hyperintense in T2 and T2*-weighted images that exhibit a crossing pattern against the surrounding hypointense dominant tissue.

We also calculated T2 relaxation times and apparent diffusion coefficients for selected regions of interest of several brain tumor biopsies. These values are shown in Table 2. T2 relaxation time and apparent diffusion coefficient regional heterogeneity allows discrimination of microstructural elements identified by histopathology. Interestingly, some microstructural elements, such as calcifications or collagen, exhibit different relative intensities and T2 values in different tumor types.

## Discussion

MRM studies on various samples have been carried out previously with resolution down to several micrometers. Many of these studies, performed on experimental models, suggest the potential applications of MRM to the study of human brain tumors [16], [19]. To our knowledge, this is the first MRM structural characterization of human brain tumor samples. We obtained MRM and correlative histopathology images from nineteen meningioma and eleven glioma human biopsies. Our protocol enables MRM previous to histopathology of the brain tumor sample, without altering the established clinical routine. These results show that the spatial organization of microstructural elements within the biopsy is preserved despite the agarose matrix and the high magnetic field. The correlation between histopathology and MRM images is critical for interpretation of results and for the success of clinical applications of the technique. The findings reported here provide a new and unique microstructural view of intact human brain tumor tissue. MRM gives detailed information about morphology, water diffusion, molecular mobility and physicochemical properties of the different elements observed. This provides an added value, with potential diagnostic implications, including better slice orientation selection and therefore less sampling errors in histopathology analysis, over conventional anatomical pathology procedures. At this point, our approach and results allow the identification of specific tissue types and pathological features in unprocessed tumor samples.

The MRM images of human brain tumor biopsies show high microstructural detail. The agarose matrix provided semi-rigid support for the sample allowing better image definition in the sample borders. The use of an agarose matrix improves the image quality mainly because of three reasons. First, it supports the sample away from the bottom of the tube allowing better sample positioning in the z-axis. Second, it minimizes the variations in magnetic susceptibility between the sample and its surroundings. Third, it reduces potential sample motion artifacts by the possible gradient vibration. From a practical point of view, the agarose matrix also facilitates the field homogeneity set up process (shimming) because it reduces the global magnetic susceptibility heterogeneity of the inter-coil space. Microscopic air bubbles trapped inside the gel matrix may generate T2* artifacts in some images due to the abrupt difference in magnetic susceptibility between air and agarose. However, they are usually at least 2 mm away from the sample and do not disturb the morphological examination of the MRM images.

Correlative MRM and histopathology allowed for quantification of MR image intensity and microstructural properties (i.e. ADC and T2 relaxation time) at specific region of interest defined by a pathologist. The marking of the fixed tissue sample with a wedge at the topmost edge allows the image registration between MRM and histopathology slices. In the scanner, the MRM virtual slicing geometry is selected by keeping this wedge at the top of the slice and producing the largest tissue section area. Histopathology slicing orientation is carefully selected to keep these very same restraints. The resulting images allow direct comparison between MRM and histopathology. Although in some of the individual images only some elements were detectable based on MRM intensities, the combination of different experiments (T1-, T2-, T2*- weighted images, proton density images and diffusion weighted images) provided characteristic patterns, detailed in Table 2, that allow the identification of many elements of pathological interest. In a few years, clinical in vivo MRI at high fields may become possible. The correlation between MRM patterns and histopathological features in brain tumors will probably facilitate the interpretation of high field in vivo MRI in patients. Our study establishes the basis of this correlation opening new potential clinical applications of MRM. On the other hand, the three-dimensional detection, before tissue processing, of microstructural elements of interest in the intact biopsy offers a unique opportunity for the pathologists to select the slicing orientation that provides the most informative tissue sections.

The only test needed to diagnose a brain tumor is cranial MRI. However, the gold standard for typing and grading brain tumors is histopathology on the basis of the most malignant area identified, according to the WHO system [1]. Accurate pathological grading is essential because it defines treatment and prognosis. Correlation between radiological and histopathological findings is of paramount importance. Bringing together MRI and histopathology of brain tumors may greatly benefit decision taking in brain tumor management and may open new possibilities for accurate in vivo tumor grading. In this work, we identified and characterized by MRM several brain tumor microstructural features critical for histopathology diagnosis and grading. Presence and extension of necrotic foci, for example, are clear markers of malignancy. In astrocytic tumors, vascular proliferation is a marker used for tumor grading. On the other hand, vascular proliferation and necrosis are never present in low grade astrocytoma. Tumor infiltration is also a marker of potential relapse. In our results, the detection of differential MRM properties in peritumoral regions correlates with lack of infiltration in the histopathological study. In particular, non-infiltrated peritumoral regions exhibit lower ADC values (see Figure 2E) as the possible result of compacted extracellular space in response to the tumor growth. Other features detected here have also been correlated with brain tumor type or grade. Some studies suggests that calcification in different types of brain tumors contain different proportions of calcium, phosphorus, magnesium, sulfur, potassium, and sodium [20]. This may explain why the T1 and T2 intensities of calcifications vary between samples.

The potential usefulness of the characterization of collagen pattern and distribution for cancer diagnosis has been suggested for many years [21]. Bolonyi studied the potential role of differential reticulin or type III collagen distribution for pathological classification of brain tumors [22]. More recent works reported that type IV collagen patterns in brain tumors may be used for differential diagnosis between meningiomas and neurilemmonas [23]. However, some studies suggest that silver impregnation techniques for reticulum fibers may have low value for determining the type III collagen molecular properties [24]. The determination of three-dimensional patterns and molecular mobility properties of collagen fibers and bands would add some valuable information in the interpretation of conventional collagen stains. In glial tumors, collagen fibers are generally limited to perivascular regions. Additionally, collagen expression and distribution correlate to vascular patterns, cell invasion and tumor microstructure [25], [26]. On the other hand, meningiomas exhibit a collagen arrangement that is to a great extent independent of vessels. We also observed different collagen patterns that exhibit minor variations in MRM signal intensity, T2 values and ADC values, suggesting different molecular microstructure and properties (Table 2). The reasons for these differential molecular properties are very diverse. Molecular mobility is partially restricted in an anisotropic manner within the fibers. For example, an increase in the directionality of the water diffusion tensor may result in artificially larger ADCs [27]. This may produce contrary effects in different images. Overall, it seems that these molecular properties detected by MRM may reflect the distinct origin of the different collagen fibers and bands (basement membranes in normal brain, specific vascular patterns, or epithelial linings). However, more specific studies on this particular subject are needed for stronger correlations between the different collagen patterns and their MRM properties. Overall, this information on collagen three-dimensional pattern and molecular properties suggests an important added value for MRM in the diagnosis of brain tumors.

T2 values and relative intensities in T1- and T2-weighted images varied among the different structural elements in the different tumor types. The potential explanations for this are far from simple. Previous studies have shown that the presence or the release to the extracellular matrix of different molecules (deoxyhemoglobin, hemosiderin, methemoglobin, iron compounds, etc) have strong and complex effects in tissue MRM relaxation rates [13], [28]. However, this does not preclude the association of particular parameters to specific pathological findings. The relaxation times and MRM relative intensities reported here may differ to those in vivo. Physical and physiological conditions, such as temperature, diffusion, perfusion, oxygenation, and biomolecular structure directly affect MRM parameters [29]. In addition, fixation causes a number of changes in the cross-linking of proteins that affects water motility and T2 relaxation [30], [31]. Therefore, the comparison between these results and in vivo measurements require some caution. In any case, this study and the intra-sample comparative values reported here may serve as bases for future high field MRI studies of brain tumor tissue.

For the first time, MRM and correlative histopathology of brain tumor biopsies yield high detail structural information. MRM provides detailed information about physicochemical properties of the different tissues analyzed, which constitutes an added value over conventional histopathology observations. The use of MRM to study human brain tumor samples may have a three-fold benefit with potential diagnostic implications. First, it may provide new microstructural information on brain tumors for better classification and characterization. Second, it may help in the interpretation of MRI clinical images in terms of cell biology processes and tissue patterns. And third, and most importantly for clinical diagnosis purposes, as it provides 3D information in intact samples, it may help in selecting a preferential orientation for the histopathology slicing which contains most of the informative elements of the biopsy.

Brain tumor MRM images show high heterogeneity both inter and intra-sample. The combination of different MRM images and R2 and ADC maps provides unique patterns for the identification of specific microstructural elements. We report MRM parameters for different pathological features representative of brain tumor type and grade, like necrotic foci, hemorrhage, vascular pattern, collagen bands and calcifications, among others. The techniques and analysis presented here provide the basis for standardized MRM examinations of brain tumor samples.

The MRM analysis of meningioma and glioma samples revealed microstructural details of these tumors, which added some structural information for clinical MRI images interpretation. We believe that our results have also potential pathobiological significance as MRM is capable of exploring the tissue sample before processing.
